# Stretchable Microelectrode Arrays with Microneedles for Reliable Electrophysiological Recording of Human Heart and Brain Organoids

**DOI:** 10.1002/advs.75778

**Published:** 2026-05-20

**Authors:** Eunyoung Jang, Saewoon Shin, Seul‐Gi Lee, Kiup Kim, Yoojeong Kim, Jun Sun, Il‐Joo Cho, Joseph A. Gogos, Jong‐Chan Park, C‐Yoon Kim, Hyunjoo J. Lee

**Affiliations:** ^1^ School of Electrical Engineering Korea Advanced Institute of Science and Technology (KAIST) Daejeon Republic of Korea; ^2^ Department of Biophysics Sungkyunkwan University Gyeonggi‐do Republic of Korea; ^3^ Institute of Quantum Biophysics Sungkyunkwan University Gyeonggi‐do Republic of Korea; ^4^ College of Veterinary Medicine Konkuk University Seoul Republic of Korea; ^5^ Department of Convergence Medicine College of Medicine Korea University Seoul Republic of Korea; ^6^ Department of Anatomy & Brain Korea 21 Plus Program for Biomedical Science College of Medicine Korea University Seoul Republic of Korea; ^7^ Mortimer B. Zuckerman Mind Brain Behavior Institute Columbia University New York New York USA; ^8^ Stavros Niarchos Foundation Center for Precision Psychiatry and Mental Health Columbia University New York New York USA; ^9^ Department of Biopharmaceutical Convergence Sungkyunkwan University Suwon Republic of Korea; ^10^ Department of Metabiohealth Sungkyunkwan University Gyeonggi‐do Republic of Korea; ^11^ KAIST Institute for NanoCentury (KINC) Daejeon Republic of Korea; ^12^ Department of Bio and Brain Engineering Korea Advanced Institute of Science and Technology (KAIST) Daejeon Republic of Korea

**Keywords:** cerebral organoid, heart organoid, microneedle, organoid, PEDOT:PSS, recording, stretchable multi‐electrode array

## Abstract

Real‐time, non‐destructive monitoring of electrophysiological dynamics of 3D organoids is imperative for advancing disease modeling and high‐throughput drug screening. However, obtaining continuous, reliable signals remains difficult due to the destructive nature of penetrating probes and the unreliable contact issue prone to surface recordings. Here, we present a stretchable 3D microelectrode array with microneedles (3D MN‐sMEA) fabricated via a scalable wafer‐level stud‐bump bonding process for minimally destructive and stable monitoring. We achieve high‐fidelity, reliable electrophysiological recordings of both human iPSC‐derived heart and cerebral models. Compared with 2D and 3D planar microelectrodes, 3D microelectrodes with microneedles achieve a higher signal‐to‐noise ratio and greater long‐term recording stability. Furthermore, quantitative pharmacological profiling validates its ability to enable precise drug screening. By combining scalable manufacturing with flexible, tissue‐compliant interfaces, our approach enables stable, minimally invasive, and long‐term electrophysiological monitoring of 3D organoids for scalable disease modeling and drug discovery.

## Graphical abstract

A stretchable 3D microelectrode array with microneedles (3D MN‐sMEA) enables stable, minimally destructive electrophysiological monitoring of 3D organoids. The wafer‐level stud‐bump bonding process facilitates high‐fidelity recordings of heart and cerebral organoids. The tissue‐compliant interface enhances scalable disease modeling and drug discovery by combining structural flexibility with minimally invasive anchoring electrodes.

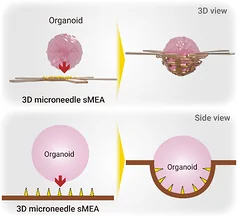

## 1 Introduction

Organoids are rapidly emerging as pivotal in vitro models that recapitulate the complex physiology of human organs [1, 2, 3]. The rapid growth in organoid research is largely attributed to the intrinsic properties of organoids, which are capable of differentiating into various functional cells. Moreover, the multicellular characteristics enable the monitoring of developmental processes within specific organs [4]. Of the various methods of organoid analysis, electrophysiological recording from electrogenic organoids, such as those of the brain and heart, using microelectrode arrays (MEAs) offers a distinct advantage with its capability for long‐term monitoring in a single organoid. For example, in brain organoids, MEA‐based electrophysiological measurements enable the assessment of functional connectivity through multichannel recordings of single spikes, synchronized bursts, and spatial mapping [5]. This technique also facilitates the quantitative analysis of organoid maturation by tracking the evolution of network activity over time [6, 7, 8]. For heart organoids, in addition to monitoring mechanical beating, electrophysiological measurements are indispensable. Parameters such as depolarization, repolarization, and conduction velocity serve as critical indicators of maturation and drug response [9, 10, 11].

Despite their broad utility, conventional MEAs exhibit significant limitations when interfaced with 3D organoids, such as shape mismatch and inability to measure intra‐organoid electrophysiology. Planar MEAs, often fabricated using standard microfabrication techniques, offer the key advantage of high‐density integration with up to thousands of electrodes. However, because the microelectrodes are often debossed below the surface of the substrate, achieving stable contact with organoids that are suspended in the culture medium is challenging without inducing deformation of the organoids through an external structure [12]. A further limitation is that their planar configuration restricts recordings to surface‐level electrical activity [13]. On the other hand, penetrating microelectrodes have been developed to access specific inner tissue layers that remain inaccessible during surface‐level recording [14, 15, 16] and thus offer a strategy to bypass the signal‐attenuating outer layers [17]. However, the rigid substrate of the conventional microelectrode arrays creates a significant mechanical mismatch with the soft organoid tissue. This mismatch induces repetitive mechanical stress and cell detachment‐induced apoptosis, which is detrimental to long‐term measurements. This problem is particularly exacerbated in models with continuous dynamic deformation, such as heart organoids.

To overcome these limitations, low‐modulus materials‐based penetrating electrodes on rigid surfaces and flexible substrates covering the 3D structure of organoids with planar electrodes were proposed. For instance, penetrating electrodes fabricated from liquid metals [18, 19], such as Eutectic Gallium‐Indium (EGaln), exhibit tissue‐like low elastic moduli and low impedance. These properties minimized interfacial damage upon insertion and have been demonstrated in vivo to provide reliable, high‐fidelity signals long‐term, even under dynamic deformations such as myocardial contraction and relaxation. The potential of this approach has also been validated for long‐term recordings from both brain and heart organoids. However, achieving high coverage envelopment of the 3D organoid structure has remained difficult, often due to the intrinsic stiffness and limited geometric conformability of the device substrate. To address this challenge, other approaches improve surface coverage using flexible substrates [9, 20] and by achieving flexibility through structural engineering [21, 22]. While these high‐coverage designs enable monitoring of the macro‐scale dynamics over a large portion of the organoid area, their planar electrode surface limited their sensing capabilities due to physical gaps between the electrode and tissue. Moreover, a critical limitation of non‐penetrating surface interfaces is their unstable contact with the organoid surface. Without anchoring elements, the solid contact between the microelectrode and the organoid is not guaranteed due to media presence and can change over time due to movements of the organoid and media. Recent advances in stretchable mesh electrodes utilized graphene microneedles to interface with irregular 3D nervous tissues [23]. However, the elastic nature of TPU‐graphene microneedles introduced variability in electrode‐tissue contact localization and signal consistency across repeated experiments. These limitations necessitate a platform that ensures robust anchoring of 3D organoids while preserving the structural and mechanical integrity of the electrodes.

**FIGURE 1:**
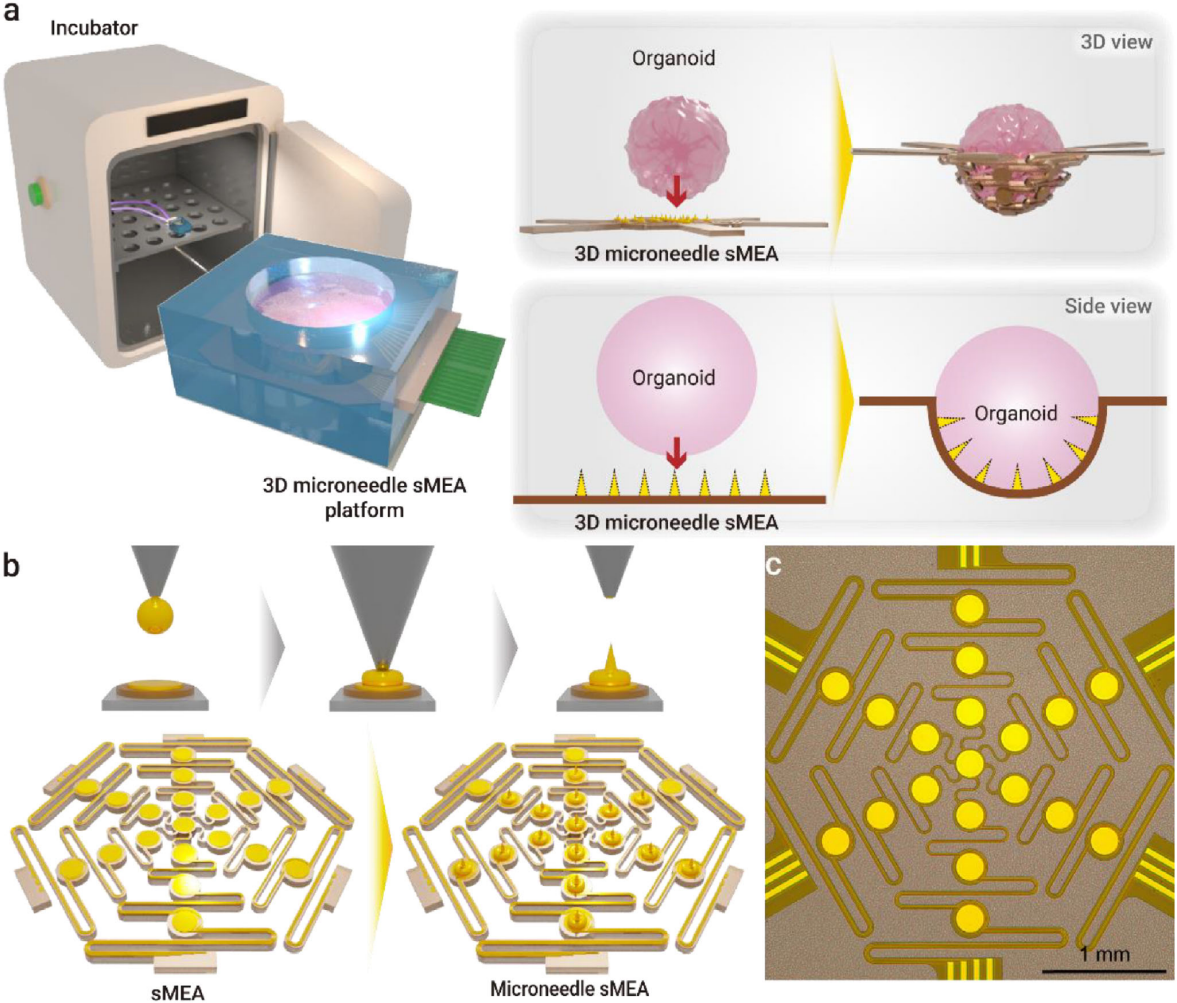
Design and fabrication of the 3D microneedle stretchable microelectrode array (3D MN‐sMEA) platform. (a) Schematic illustration of the experimental setup, including the packaged MN‐sMEA platform inside the incubator, 3D conformal interfacing of organoid using serpentine structures, and improving the contact with the outer layer of organoid using microneedles. (b) Schematic illustrations of microneedle formation on the flexible microelectrode array via gold stud bump process. (c) Optical image of the fabricated flexible microelectrode array (top view).

To simultaneously overcome the geometrical limitation of rigid penetrating MEAs and the contact instability of flexible MEA's electrodes, we propose a novel hybrid 3D microneedle stretchable microelectrode array (3D MN‐sMEA) that combines a mechanically flexible serpentine structure with microneedles (Figure 1a). The device integrates microneedles, which are readily formed using stud bump bonding (Figure 1b), onto a structurally flexible substrate developed via a MEMS process (Figure 1c). Specifically, the interconnects utilize a serpentine design with bridge structures that effectively dissipate mechanical stress, which allows the array to stretch and wrap conformally around the curvilinear surfaces of organoids without structural failure. This design ensures high‐coverage recording from within the organoid while minimizing mechanical damage during dynamic deformation. Unlike needle‐free planar stretchable microelectrode arrays (sMEAs), which are limited to surface signals and are impeded by the high‐resistance outer layers of organoids, the integrated microneedles minimally penetrate the tissue, enabling the acquisition of high‐fidelity signals by bypassing the high‐resistance layers. Furthermore, the 3D MN‐sMEA exhibits long‐term stability compared to conventional 2D rigid MEAs, which suffer from rapid signal degradation and tissue deformation. The 3D configuration maintains robust electrode‐tissue contact by mechanical anchoring, preserving a high signal‐to‐noise ratio (SNR) over extended periods. We validate these capabilities through stable electrophysiological recordings in both heart and brain organoids.

## 2 Results and Discussion

### 2.1 Design and Structure of 3D MN‐sMEA Platform

**FIGURE 2:**
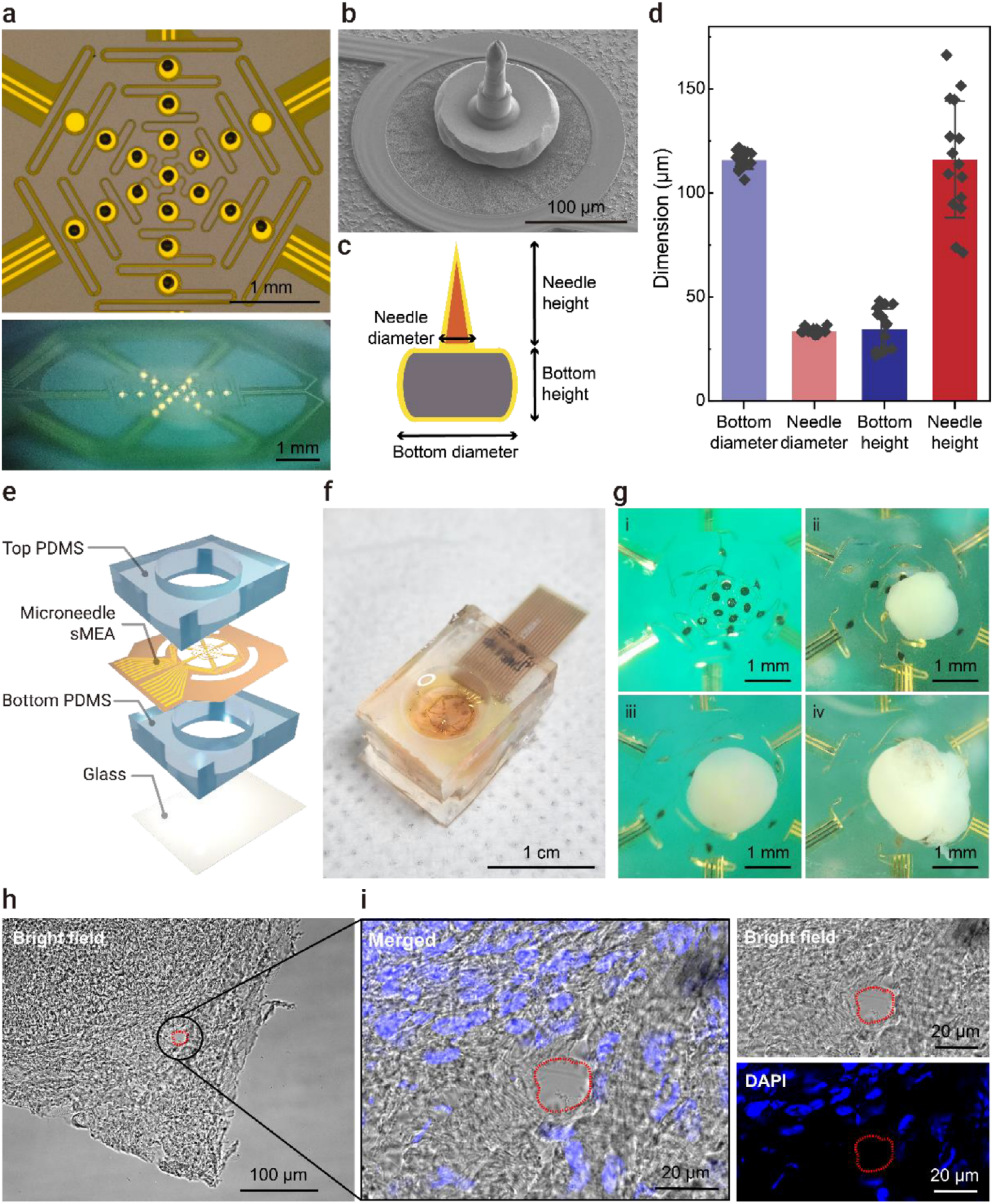
Structural characterization of the microelectrode array with microneedles. (a) Top‐view (top) and tilted‐view (bottom) optical images of the microneedle microelectrode array. (b) Tilted‐view SEM image of a single Au microneedle fabricated on a microelectrode via the stud bump process. (c) Schematic illustration showing the definition of the dimensional parameters of the microneedle. (d) Quantification of the microneedle dimensions, including bottom diameter, needle diameter, bottom height, and needle height. (e) Exploded‐view schematic of the multilayered device, comprising a glass substrate, bottom and top PDMS chambers, and the MN‐sMEA. (f) Photograph of the fully packaged platform. (g) Magnified photographs (i‐iv) demonstrating its capability to accommodate organoids of varying sizes (∼1 to 3 mm). (h) Bright‐field and (**i**) fluorescence microscopy images of a cerebral organoid confirming the penetration of a microneedle tip.

The stretchable MEA (sMEA), which serves as the foundation of the device, was fabricated with a serpentine structure on a polyimide (PI) substrate using MEMS processes adopted from a previous study to ensure structural flexibility [22]. To achieve stable signal measurements, the microneedles were individually formed on the 16 planar Au microelectrodes of the sMEA using a stud bumping process. While the stud bumping process is inherently a serial process, it offers strategic advantages such as material homogeneity with the Au microelectrodes and rapid geometric tunability without the need for specialized masks or molds. For the formation of the stud bumps, an Au wire, the same material as the microelectrodes, was utilized. The process successfully formed needle‐like structures on the PI‐based microelectrodes fabricated on a silicon substrate (Figure 2a, Figures S3 and S4). The resulting structure consists of two parts: a wide‐diameter base, which was formed as a result of gold ball compression during stud bumping, and a needle‐shaped tip extending from the base (Figure 2b,c, Figure S5). When the stud bumping was conducted under identical conditions, the average heights of the base and the needle of 15 microelectrodes were 34.48 and 116.14 µm, respectively, which resulted in a total calculated height of 150.52 µm (Figure 2d). The mechanical robustness of the stud‐bump bonded Au microneedles was qualitatively verified through handling and experimental procedures. The microneedles remained intact during transfer processes and repeated contacts with organoids during positioning. Finally, to reduce the electrochemical impedance of the 3D MN‐sMEA, a biocompatible PEDOT:PSS layer was uniformly electrodeposited.

The final 3D MN‐sMEA was packaged into a microwell by assembling it with upper and lower PDMS chambers and a glass substrate (Figure 2e,f, Figure S6). The optimized serpentine structure allows the device to conform to the spherical surface of the organoid without mechanical stress, providing stable support. To validate the capability of the packaged microneedle stretchable MEA (MN‐sMEA) to accommodate organoids of various sizes, organoids with diameters ranging from approximately 1 to 3 mm were placed on the 3D MN‐sMEA (Figure 2g). The serpentine structures naturally stretched to stably support the spherical surface without imposing any significant external pressure on the organoids of various sizes, and thus, the intrinsic 3D morphology of the organoids was preserved (Figure S7).

Next, to observe the insertion characteristics of the microneedle on the stretchable MEA, we placed a cerebral organoid on the device for approximately 1 h and examined the surface (Figure 2h). DAPI staining was performed to label cell nuclei around the needle penetration site (Figure 2i). The observation confirmed the formation of pores with a diameter of roughly 18 µm on the surface. This indicates that the wide base of the microneedle, with an average diameter of 113.33 µm, did not fully penetrate the tissue. Instead, a portion of the tapered needle tip with a diameter of approximately 34.26 µm partially penetrated the organoid.

### 2.2 Electrochemical Impedance of the 3D MN‐sMEA

**FIGURE 3:**
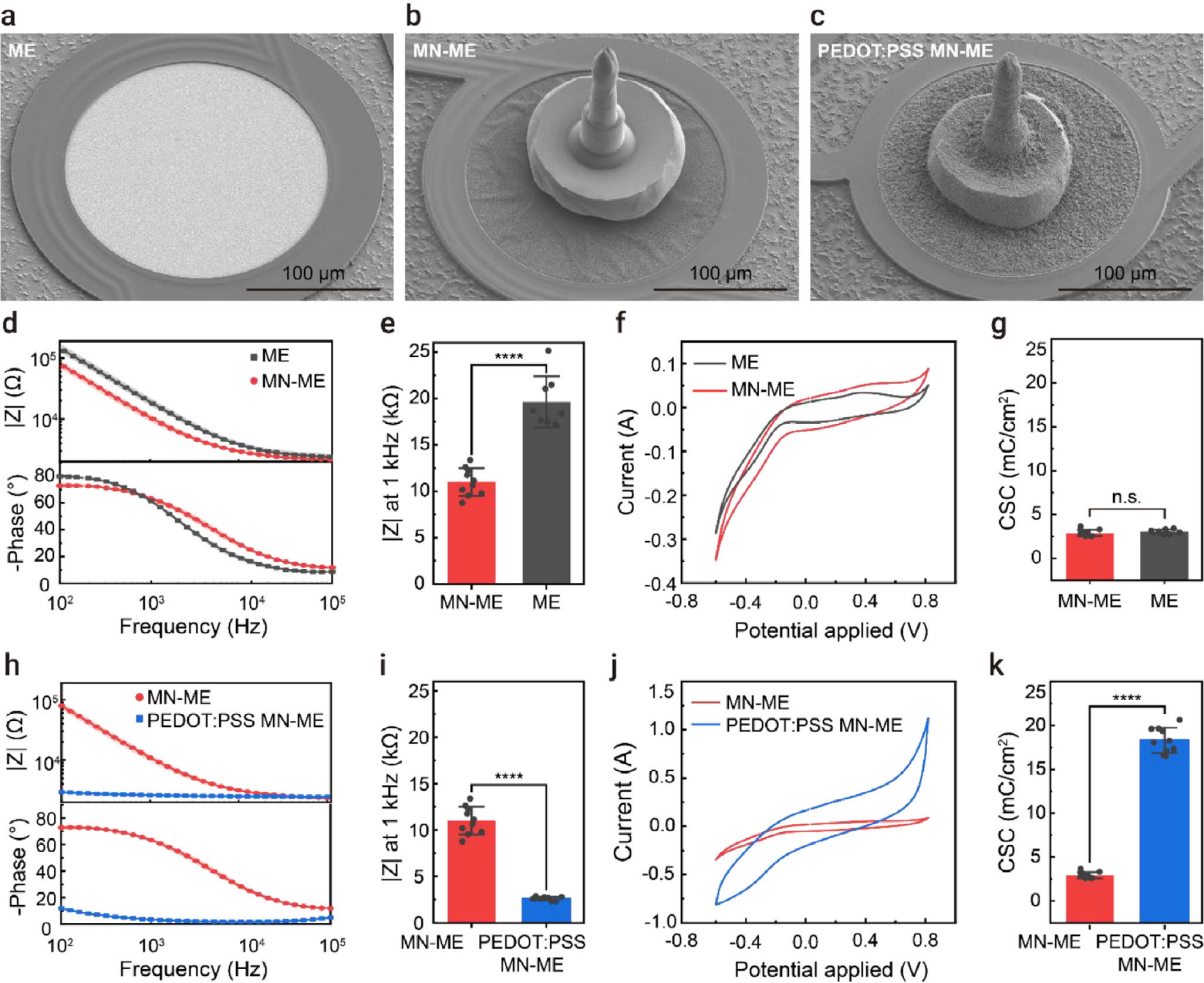
Fabrication, characterization, and electrochemical performance of the microelectrode array with microneedles. SEM images of the (a) planar microelectrode (ME), (b) microneedle microelectrode (MN‐ME), and (c) PEDOT:PSS electrodeposited microneedle microelectrode (PEDOT:PSS MN‐ME). Comparison of electrochemical properties between planar ME and MN‐ME showing (d) electrochemical impedance spectroscopy (EIS), (e) impedance magnitude at 1 kHz, (f) cyclic voltammetry (CV), and (g) charge storage capacity (CSC). Evaluation of PEDOT:PSS electrodeposition on MN‐ME showing (h) EIS plots, (i) impedance magnitude at 1 kHz, (j) CV, and (k) CSC, comparing MN‐ME before and after coating. Data are presented as mean ± SD. ^****^ *p* < 0.0001; n.s., not significant.

We compared the electrochemical characteristics of three types of microelectrodes: planar microelectrode (ME), microelectrode with microneedle (MN‐ME), and PEDOT:PSS electrodeposited microelectrode with microneedle (PEDOT:PSS MN‐ME) (Figure 3a–c). First, the electrochemical performance of microelectrodes with microneedles was compared with that of the planar electrodes by evaluating the electrochemical impedance spectroscopy (EIS) and charge storage capacity (CSC) (Figure 3d–g). A clear decrease in the electrochemical impedance was observed, where the impedance at 1 kHz decreased by approximately 44% from 19.6 kΩ (planar) to 11.0 kΩ (microneedle) (Figure 3e). This reduction is attributed to the increased active surface area of the microneedle electrodes. On the other hand, the CSC values were comparable, which indicates that the charge‐storage capability per unit area of the original gold microelectrodes was maintained after microneedle formation (Figure 3g).

Next, the EIS and CSC of the microelectrodes with microneedles were compared before and after PEDOT:PSS deposition (Figure 3h–k). We deposited a PEDOT:PSS layer to improve both the electrochemical impedance and CSC characteristics. Prior to comparison, we first confirmed the deposition was successful by observing the structural changes. When PEDOT:PSS was deposited under the same conditions as for the planar microelectrodes, the surface exhibited similar structural characteristics (Figure S8). PEDOT:PSS deposition reduced the electrochemical impedance by 76% from 11.00 to 2.62 kΩ at 1 kHz (Figure 3i). As expected, the CSC improved by approximately 6.3‐fold, from an average of 2.91 mC/cm^2^ before deposition to 18.30 mC/cm^2^ after deposition (Figure 3k). Planar microelectrodes required a thick PEDOT:PSS coating to form a protruding structure for improved contact, but at the risk of structural instability due to weak adhesion. In contrast, the microneedle electrodes maintained the protruding morphology and thus required only a thin PEDOT:PSS coating. This configuration ensured stability, resulted in lower impedance, and enhanced CSC. Furthermore, the electrochemical stability of the PEDOT:PSS deposition was confirmed by monitoring the impedance magnitude at 1 kHz in a physiological environment (PBS at 37°C) for 10 days, which showed no significant variation (Figure S9).

### 2.3 Enhanced Signal Quality and Stability in Human Heart Organoid (hHOs) using the 3D MN‐sMEA

The proposed 3D microneedle sMEA preserves the native structure of 3D organoids while achieving superior electrode contact, which enables highly reliable measurement of the intrinsic functional properties. To demonstrate these advantages, we compared the 3D MN‐sMEA with 2D MN and 3D‐sMEA with two hypotheses. First, it was hypothesized that the 3D MEA configuration would enhance the physiological stability of the organoids compared to a 2D planar MEA, as 3D shape is preserved. Second, it was postulated that within the 3D structure, the recording method using microneedles would provide superior signal quality over the planar recording method.

**FIGURE 4:**
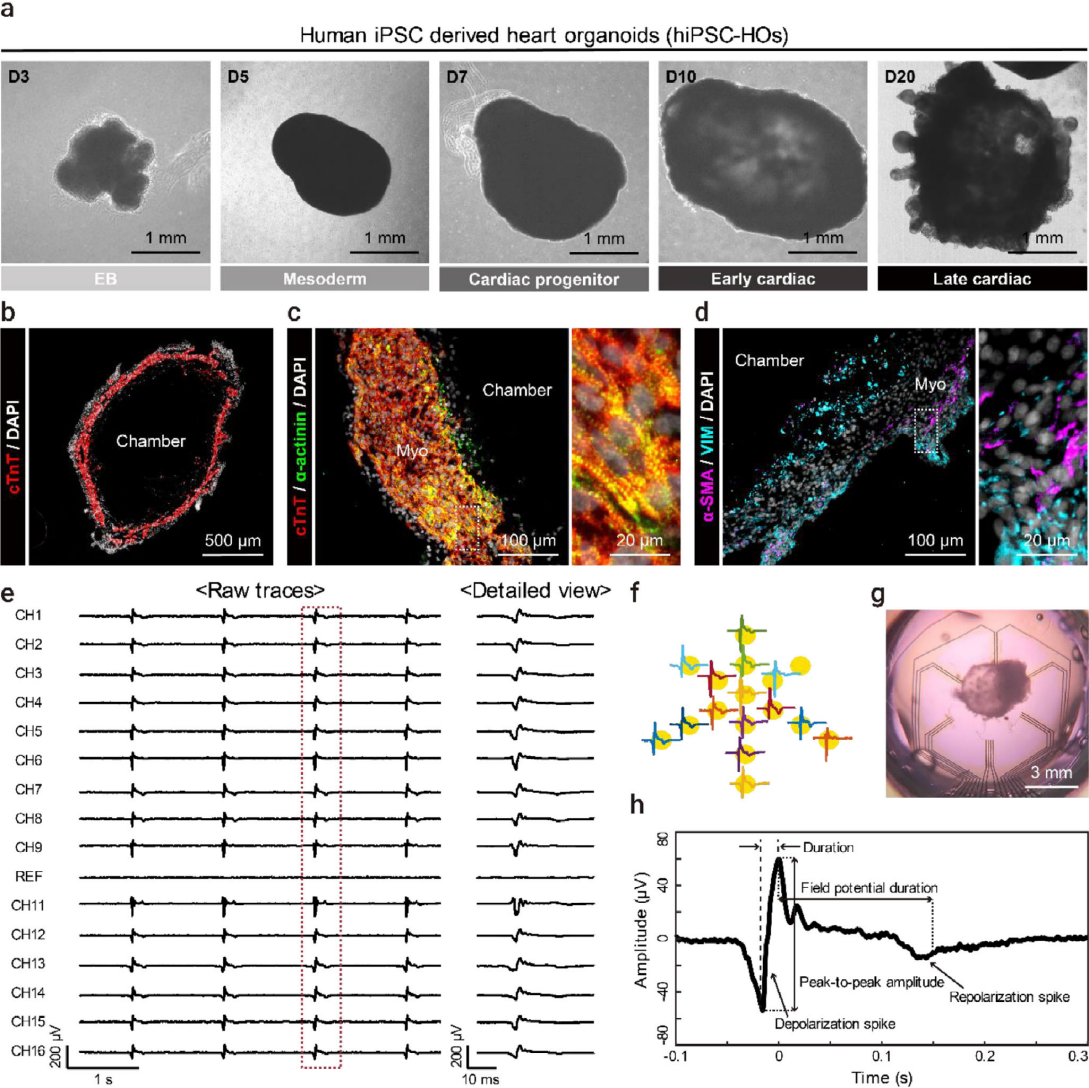
Measurement of electrophysiological signals of human heart organoids (hHOs) using the 3D MN‐sMEA. (a) Generation of human iPSC‐derived heart organoids. Immunofluorescence staining for (b) cTnT (CMs), (c) α‐actinin (z‐disc), and (d) α‐SMA (SMCs), VIM (Fbs) to identify cardiac components in hiPSC‐HOs. (e) Raw traces of field potentials recorded from 16 channels. (f) Schematic showing the placement of 16 electrodes. (g) An optical image of an hHO positioned on the 3D MN‐sMEA. (h) A representative field potential waveform illustrating key quantified parameters: depolarization spike, repolarization spike, peak‐to‐peak amplitude, duration, and field potential duration.

Prior to testing the hypothesis, we first verified the capability of the 3D MN‐sMEA to reliably record field potentials from 20‐day‐matured iPSC‐derived heart organoids (hHOs) (Figure 4a–d). Specifically, the combination of rigid needles and a flexible substrate is uniquely advantageous for monitoring contractile HOs, as it ensures high electrode‐tissue stability and reliable signal acquisition during continuous mechanical cycles while minimizing bulk tissue deformation (Table S1). hHOs were generated through stepwise differentiation and maturation using previously reported protocols (Figure 4a). Within the hHOs, a myocardium‐like region with band‐shaped expression of the cardiomyocyte (CM)‐specific marker cTnT formed around the chamber, and clear sarcomeric structures in self‐organized CMs, along with other cardiac constituent cell types, were also observed (Figure 4b–d). For electrophysiological recordings, the hHOs were placed onto the packaged 3D MN‐sMEA platform. Periodic field potentials were successfully detected across all recording channels except the reference channel, which demonstrated enhanced electrode–tissue contact (Figure 4e–g). Recording from all 15 channels enabled signal acquisition over a larger region of the organoid, which will facilitate the tracking of functional changes induced by various experimental factors. The high signal‐to‐noise ratio (SNR) of the recorded field potentials further allowed quantitative analysis of key electrophysiological features, including peak‐to‐peak amplitude, duration, and field potential duration (Figure 4h). The field potential duration (FPD) is the time interval from the onset of depolarization to the completion of repolarization and serves as a critical electrophysiological metric for assessing the cardiac cycle and cardiotoxicity [32]. In this study, the baseline FPD of hHOs was measured at 147.69 ms. We confirmed that the measured FPD values are well within the physiologically relevant range (approximately 100–300 ms) reported in previous studies using various heart organoid models or hiPSC‐derived cardiomyocyte MEA systems [9, 11, 22, 32].

**FIGURE 5:**
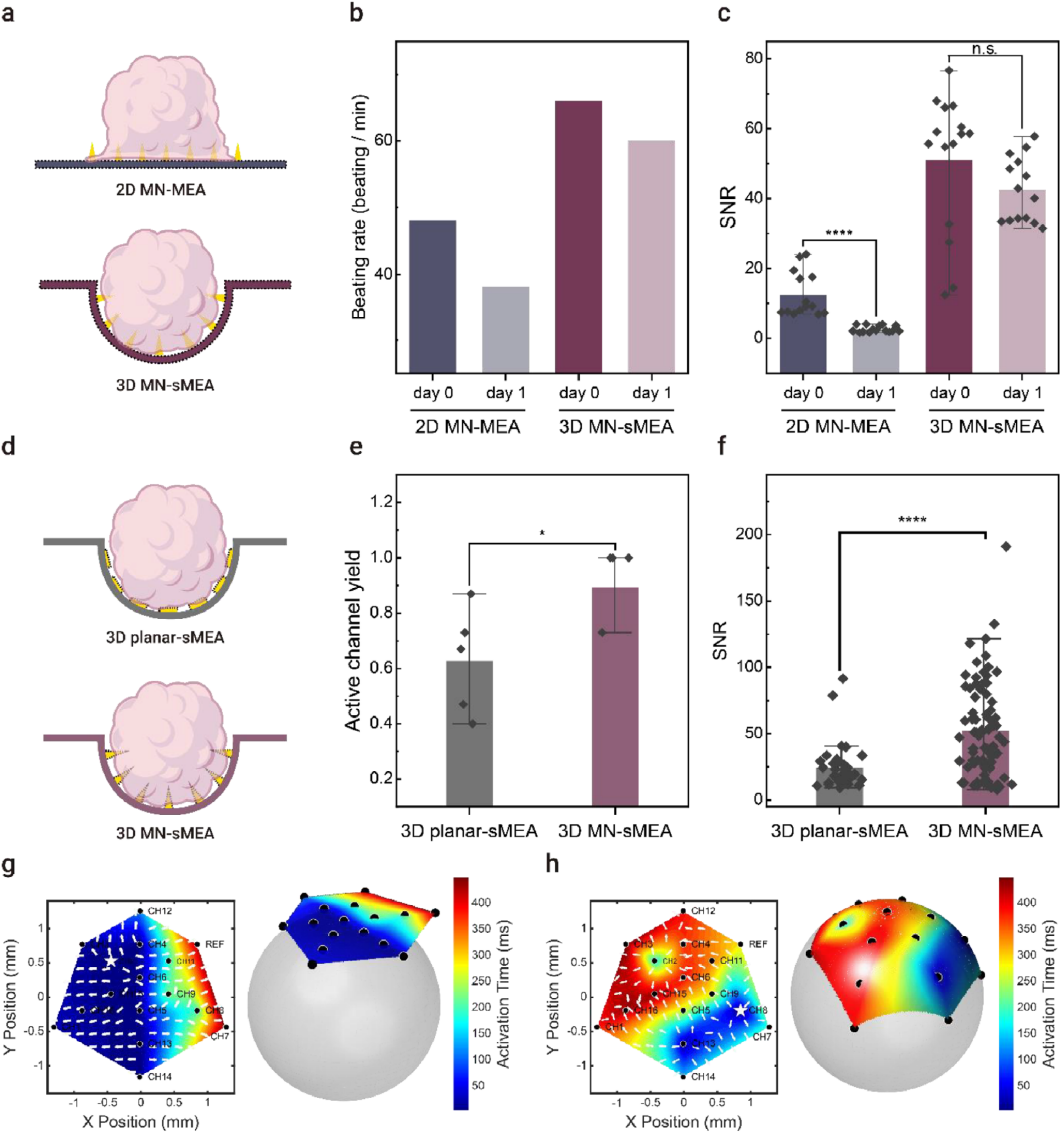
Enhanced signal quality and stability in human heart organoids (hHOs) using the 3D MN‐sMEA. (a) Schematic illustrations of experimental setups for 2D MN‐MEA and 3D MN‐sMEA. (b) Comparison of beating rates at day 0 and day 1, and (c) signal‐to‐noise ratio (SNR). (d) Schematic illustrations of 3D planar‐sMEA and 3D MN‐sMEA to compare the surface and intra‐organoid recording. (e) Active channel yield and (**f**) SNR comparison between 3D planar‐sMEA and 3D MN‐sMEA. Data are presented as mean ± SD. ^*^ *p* < 0.05, ^****^ *p* < 0.0001; n.s., not significant. (g) Field potential activation map obtained from the 2D MN‐MEA. The right panel illustrates the planar projection map with a spherical organoid phantom. The white star indicates the earliest activation site where the spontaneous electrical signal originated within the organoid. (h) Field potential activation map obtained from the 3D MN‐sMEA. The right panel illustrates the volumetric propagation map with a spherical organoid phantom.

To test the first hypothesis, the field potential signals from hHOs were compared between a 2D microneedle MEA (MN‐MEA) and the 3D microneedle sMEA (MN‐sMEA) (Figure 5a). For the 2D MN‐MEA, hHO was plated and immobilized by adjusting the culture medium volume, which mimics the measurement conditions of commercial MEAs. The signals were measured immediately after plating the hHO and again after approximately 24 h. Initially, the beating rate of 2D MN‐MEA and 3D MN‐sMEA was 48 and 66, respectively. In addition, the beating rate on the 2D MN‐MEA decreased by approximately 20.83%, from 48 to 38 beats per min, whereas the rate on the 3D MN‐sMEA decreased by only ∼9%, from 66 to 60 beats per min, which confirms superior long‐term culture stability (Figure 5b). Furthermore, we compared the SNR, which was calculated by dividing the peak‐to‐peak amplitude of the field potential by the baseline amplitude [24]. The SNR of 2D MN‐MEA decreased by 78.7% while that of 3D MN‐sMEA decreased by only 17.0%, which demonstrated higher stability for 3D MN‐sMEA (Figure 5c). This performance decline can be attributed to the limited supply of nutrients and oxygen that occurs when the organoid is attached to an impermeable surface. Conversely, the 3D serpentine structure of the proposed device ensures 3D contact with the culture medium, which help maintain the physiological homeostasis of the hHO. This feature can lead to enhanced stability for long‐term measurements. In addition, our 3D electrode configuration enables the reconstruction of volumetric signal‐propagation maps that accurately reflect the physiological geometry of the organoid, whereas the conventional 2D mapping is limited to the planar‐projection (Figure 5g,h).

The second hypothesis was tested by comparing the performance of the 3D microneedle electrodes with that of 3D planar microelectrodes by analyzing the field potential signals from hHOs (Figure 5d). Five hHOs were plated on each of the 3D MN‐sMEA and 3D planar sMEA, and signals were measured approximately two min after plating. The active channel yield, defined as the proportion of the 15 electrodes that detected field potentials, was 89.2% for the 3D MN‐sMEA, which is a higher value than the 62.8% for the 3D planar sMEA (Figure 5e). Furthermore, the 3D MN‐sMEA exhibited a significantly higher SNR of 54.25, more than twice that of the 3D planar sMEA (Figure 5f). The planar microelectrodes measure signals through partial contact with the cells on the hHO surface, whereas the microneedle electrodes are partially inserted into the tissue, which capture signals more robustly. This result demonstrated the intrinsic difference between surface and minimally invasive sub‐surface recording. While the precise depth may vary depending on the stretchable nature of the substrate and the morphological irregularities of the organoids, we confirmed that the stability and depth of the needle‐tissue interface are critical factors in determining signal quality. Channels with stable and sufficient penetration consistently yielded robust electrophysiological waveforms, whereas unstable contact led to difficulties in signal detection (Figure S10). We believe that developing a method to precisely quantify and control the penetration depth will be a key to further enhancing the capabilities of this platform.

These comparative validation results demonstrate that the 3D MN‐sMEA is a promising hybrid interface that simultaneously overcomes the limitations of both 2D and 3D planar sMEAs. Our device achieves this by (1) securing long‐term culture stability through its 3D serpentine structure and (2) acquiring high‐quality internal signals via the microneedles.

### 2.4 Quantitative Analysis of Drug‐Induced Electrophysiological Responses in hHOs

**FIGURE 6:**
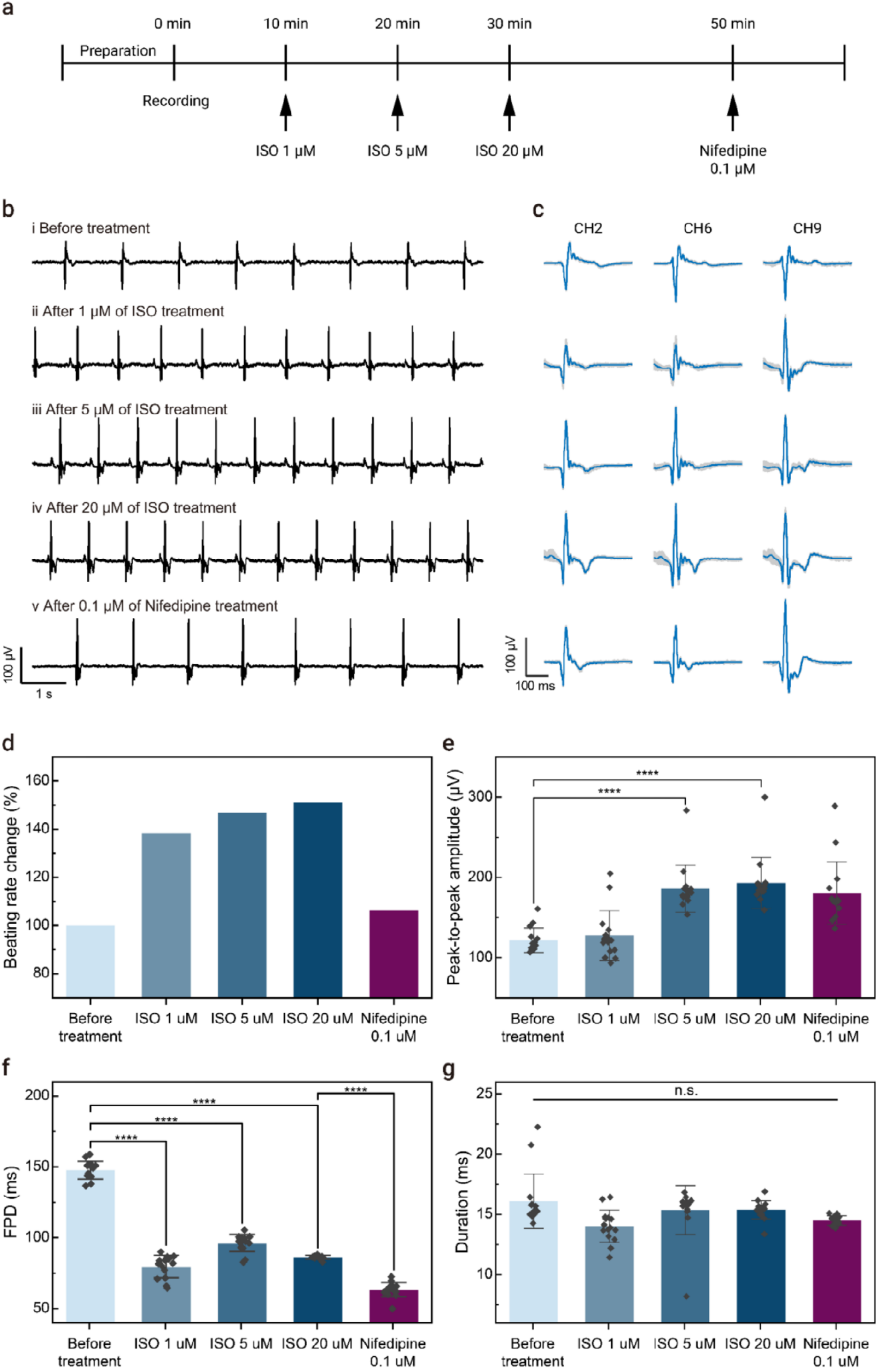
Quantitative analysis of drug‐induced electrophysiological responses in hHOs. (a) Experimental timeline illustrating the sequential administration of isoproterenol (ISO) and nifedipine. (b) Representative raw traces from a single channel showing changes in electrophysiological activity before and after treatment with ISO (1, 5, 20 µm) and nifedipine (0.1 µm). (c) Averaged field potential waveforms from representative channels (CH2, CH6, CH9) under each drug condition. Quantification of electrophysiological parameters: (d) beating rate, (e) peak‐to‐peak amplitude, (f) signal duration, and (g) field potential duration (FPD). Data are presented as mean ± SD. ^*^ *p* < 0.05, ^****^ *p* < 0.0001; n.s., not significant.

To validate the applicability of our platform for drug screening, the electrophysiological responses of hHOs to the cardioactive drugs, isoproterenol (ISO) [25] and nifedipine [26], were quantitatively analyzed. The analysis timeline was as follows (Figure 6a): after plating the hHO, signals were recorded for 10 min without any drug treatment. Subsequently, ISO was administered at concentrations of 1, 5, and 20 µm at 10‐min intervals. Twenty minutes after the final ISO administration, 0.1 µm of nifedipine was introduced to observe the changes. The real‐time signal changes following drug administration were confirmed from the raw traces (Figure 6b), and the effect of the drugs on the field potential waveform was visually identified through the averaged waveforms of a representative channel (Figure 6c). To quantitatively analyze the drug efficacy, changes in the beating rate, peak‐to‐peak amplitude, duration, and field potential duration (FPD) were extracted.

The beating rate exhibited a dose‐dependent increase as the concentration of ISO, a β‐adrenergic agonist, increased. The measurement results confirmed that upon administration of 1, 5, and 20 µm ISO, the beating rate increased to 138.30%, 146.82%, and 151.06% of the pre‐treatment rate, respectively (Figure 6d). The peak‐to‐peak amplitude showed a significant change at concentrations of 5 µm and higher compared to the pre‐treatment condition (Figure 6e). Furthermore, the FPD showed a significant decrease from 147.69 to 79.18, 95.82, and 86.09 ms upon administration of 1, 5, and 20 µm ISO, respectively. This parameter, however, did not exhibit a clear dose‐dependent trend (Figure 6f). The absence of a concentration‐dependent trend can be interpreted as the maximum shortening effect being saturated at the 1 µm concentration. Conversely, after the administration of 0.1 µm nifedipine, an L‐type calcium channel blocker, the elevated beating rate induced by 20 µm ISO was significantly reduced to 106.38% of the pre‐treatment rate. This result confirmed that the effect of ISO was antagonistically inhibited. The FPD was also significantly reduced by 26.87% (Figure 6d,f). The signal duration remained constant regardless of drug administration (Figure 6g). This platform can simultaneously track not only changes in beating rate but also subtle variations in the field potential waveform, such as peak‐to‐peak amplitude and FPD. This capability presents a powerful advantage for comprehensively evaluating the multifaceted effects of pharmacological compounds.

### 2.5 Qualitative Analysis of Drug‐Induced Electrophysiological Responses in Cerebral Organoids

**FIGURE 7:**
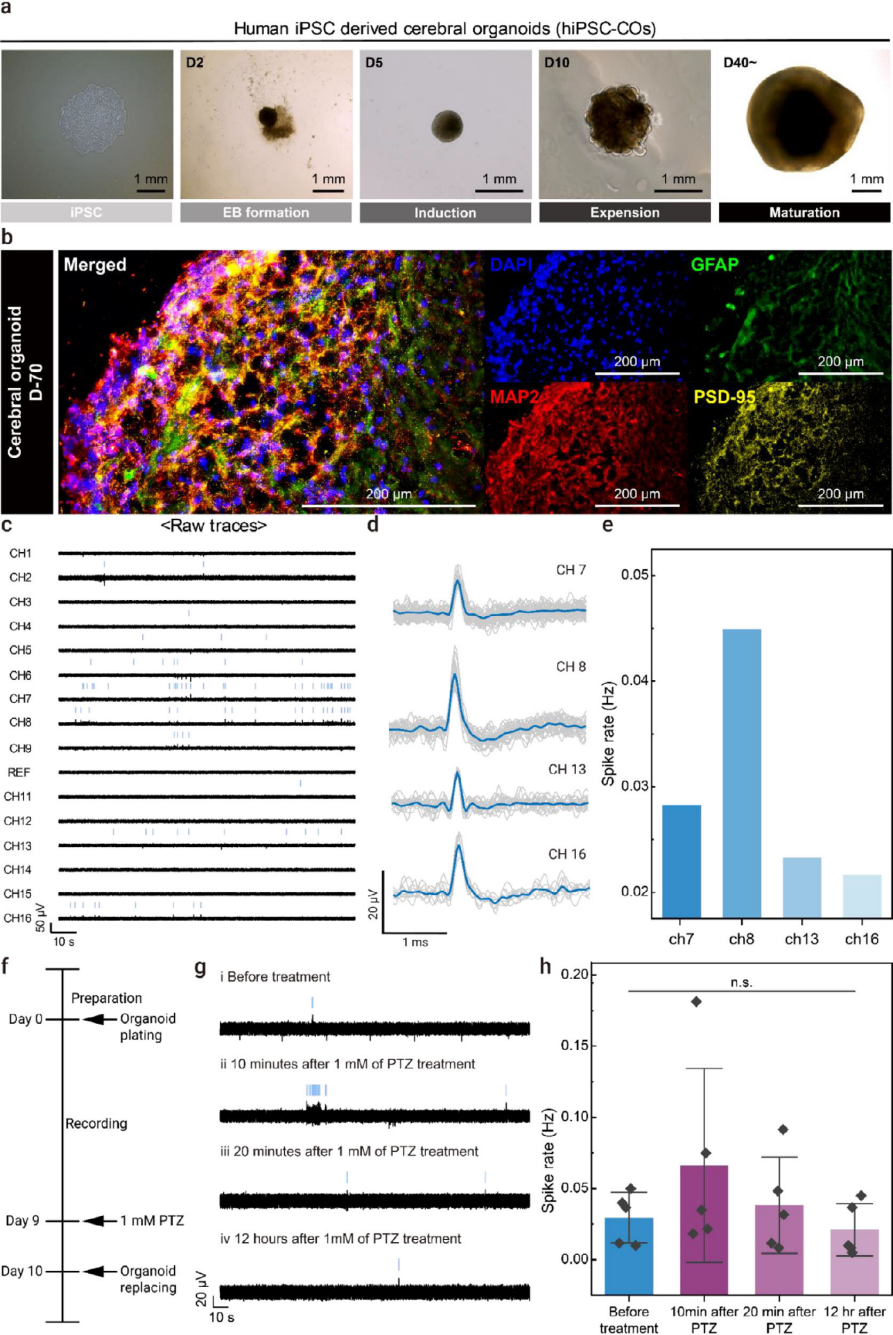
Electrophysiological recording and neuropharmacological responses of cerebral organoids using the 3D MN‐sMEA. (a) Generation of iPSC‐derived cerebral organoids. (b) Immunofluorescence images of iPSC‐derived cerebral organoids showing DAPI, anti‐GFAP (astrocyte), anti‐MAP2 (neuron), and anti‐PSD‐95 (synapse). (c) Representative raw traces of spontaneous neural activities recorded from multiple channels. (d) Averaged spike waveforms from four active channels (CH7, CH8, CH13, CH16). (e) Quantification of the mean spike rate for each active channel at baseline. (f) Experimental timeline for the administration of pentylenetetrazol (PTZ). (g) Representative raw traces showing neural activity (i) before treatment, (ii) 10 min after 1 mm PTZ, (iii) 20 min after PTZ, and (iv) 12 h after PTZ. (h) Quantification of the mean spike rate. Data are presented as mean ± SD. n.s., not significant.

To demonstrate the versatility of the platform and explore its potential for neuropharmacological applications, the drug response of functional neural networks was evaluated using cerebral organoids [27]. The mature iPSC‐derived cerebral organoids were successfully generated (Figure 7a). Immunofluorescence imaging of Day 70 cerebral organoids revealed prominent MAP2 positive, neurite rich neuronal architecture. PSD‐95 displayed abundant punctate staining throughout the MAP2 positive neuropil, consistent with widespread postsynaptic specializations and synapse formation. In addition, GFAP positive astrocytic domains exhibited a patchy distribution and were closely associated with neuronal territories, which supports a well‐developed multicellular neural tissue like organization in cerebral organoids (Figure 7b). Before drug treatment, spontaneous neural activities were measured across several channels (Figure 7c). The recorded multiple neuronal spikes were sorted, and the firing rate of the recorded signals was analyzed (Figure 7d,e).

To assess the functional response of the neural network, the organoids were treated with Pentylenetetrazol (PTZ), a GABA‐A receptor antagonist known to induce seizure‐like activity [28]. The analysis timeline was as follows (Figure 7f): signals were measured for a total of nine days at a baseline state before drug treatment. Subsequently, 1 mm of PTZ was administered, and the changes were observed after 10 and 20 min. The recovery from the pharmacological treatment was confirmed from a measurement obtained 12 h later. Qualitative observation of the changes after PTZ treatment indicated a trend of increasing network firing frequency over time (Figure 7g). Quantitative analysis revealed that the spike rate increased by an average of 58.69%, from 0.11 to 0.27 Hz, 10 min after PTZ treatment compared to the pre‐treatment baseline; however, this increase did not reach statistical significance (*p* > 0.05) (Figure 7h). The spike rate recovered to baseline levels, measuring 0.13 and 0.10 Hz at 20 min and 12 h post‐treatment, respectively.

Last, a live‐dead assay was conducted after culturing a cerebral organoid on the device for five days to confirm biocompatibility for long‐term signal recording. The results showed a high cell viability with no significant difference compared to the control group (Figure S11). The dead cell ratio remained below 0.2, which indicates the minimal cytotoxicity of the platform. This suggests that the fabricated microneedle platform did not induce cytotoxicity and did not affect the growth of the organoid, which allows for long‐term measurement studies.

## 3 Conclusion

In this study, a novel hybrid 3D microneedle stretchable microelectrode array (MN 3D‐sMEA) was successfully developed and validated to overcome the significant limitations of conventional microelectrode arrays for minimally invasive and stable electrophysiological measurements. The proposed platform uniquely integrated a structurally flexible serpentine framework, which enabled conformal contact with 3D organoids, and stud‐bumped microneedles that serve as mechanical anchors while providing direct access to viable sub‐surface tissue. By electrodepositing PEDOT:PSS, the electrode performance, including electrochemical impedance and charge storage capaitcy was significantly enhanced, which is essential for high‐fidelity signal acquisition. Through direct comparative studies, we demonstrated that the proposed 3D serpentine structural design ensures superior long‐term physiological stability compared to 2D platforms, and that the microneedle‐based interface provides a significantly higher signal‐to‐noise ratio than 3D planar microelectrodes. This validated our hypothesis that combining structural flexibility with minimally invasive anchoring electrode is a highly effective strategy for interfacing with 3D biological models. Furthermore, the versatility and quantitative analysis capabilities of the platform were validated by successful electrophysiological recordings from both human heart and cerebral organoids. We precisely tracked the dose‐dependent and antagonistic responses of the organoids to pharmacological compounds, which illustrates the potential of this platform as a powerful tool for drug screening applications. Beyond organoid monitoring, the structural flexibility of the platform and proven biocompatibility suggest high potential for in vivo applications on curvilinear biological surfaces and larger tissue systems by modifying the design while maintaining the established manufacturing workflow, which serve as a versatile tool for diverse bio‐electronic applications.

## 4 Materials and Methods

### 4.1 Fabrication of Stretchable Microelectrode Array (sMEA)

The sMEA was fabricated according to the methodology described in our previous study [22]. A 4‐µm‐thick Polyimide (PI) (GPI‐100‐A, PNS Technology, Korea) layer was spin–coated onto a silicon wafer (Namkang Hi‐Tech Co., Korea) pre‐coated with poly(methyl methacrylate) (PMMA; 950 PMMA A4, Kayaku Advanced Material Inc., Japan), followed by full imidization at 80°C, 150°C, 230°C, and 280°C for 30 min at each step. A 10 nm Ti and 100 nm gold (Au) layers were deposited with an electron beam evaporator on patterned negative photoresist (NR9‐3000PY, Futurrex Inc., USA) to form microelectrodes and signal lines. Next, a 1‐µm‐thick PI layer was spin–coated and imidized as previously explained. Copper (Cu) was deposited with a thermal evaporator on patterned positive photoresist (AZ10XT, MicroChemicals, Germany) to form a metal hard mask for body patterning. The PI layer was etched by Oxygen (O_2_)_2_ plasma. The Cu mask was removed using metal etchant (APAL‐1, Sigma–Aldrich, USA) (Figure S1).

### 4.2 Formation of Microneedles on Microelectrodes

Microneedle electrodes were formed on each individual microelectrode of the fabricated sMEA, with one microneedle per microelectrode, via stud bumping using a wire bonder (iBond5000 dual, Micro Point Pro, Israel). To begin the process, a gold wire was loaded into a capillary, and the metal pad on the bonder where the device was placed was heated. Subsequently, an electronic flame‐off (EFO) wand was activated to form a gold ball at the tip of the wire. The capillary was then lowered to the microelectrode, compressing the gold ball. Once the stud bump was formed, the capillary was raised to a predetermined position, completing the bonding process (Figure S2) [29]. The range of working holder temperature, power, time, bonding force, Z speed, tail, and ball size of the iBond5000 dual are 59°C–60°C, 2.5, 12, 5–6, 5, 5–6, and 4–5, respectively.

### 4.3 PEDOT:PSS Electrodeposition

PEDOT:PSS electrodeposition was conducted to reduce the electrochemical impedance of the microneedle‐loaded microelectrodes. Microneedle electrodes were electrically connected to a flexible PCB through ACF bonding. First, cyclic voltammetry was performed 12 times from −0.5 to 1.5 V in 0.5 m H_2_SO_4_. 0.05 m of 3, 4‐Ethylenedioxythiophene (EDOT), and 0.5 M of Poly(sodium 4‐styrenesulfonate) (PSS) were added to DI water and sonicated for 30 min. For the 3‐electrode system, the Ag/AgCl electrode and the Pt electrode were used as the reference and counter electrodes, respectively. The MN‐sMEA was dipped inside the solution with the reference and counter electrodes, and the solution was stirred during deposition. A constant current from 300 to 400 nA was applied to each microelectrode.

### 4.4 Packaging of the 3D MN‐sMEA Platform

The MN‐sMEA was released after PEDOT:PSS deposition by immersing the substrate in acetone for over 6 h to remove the sacrificial PMMA layer. After gently lifting out the MN‐sMEA from the acetone, water‐soluble polyvinyl alcohol (PVA) tape was attached on the MN‐sMEA's stretchable structure to prevent tangling of the signal lines. PVA tape attached to MN‐sMEA was then connected to the flexible printed circuit (FPC) connector. The FPC‐connected MN‐sMEA was then sandwiched between a bottom and top PDMS wells using uncured PDMS. Here, the bottom and top PDMS wells were fabricated using 3D‐printed molds, where the bottom side of the bottom PDMS well was attached to a cut slide glass. The completed platform was then gently pressed with a 500‐g weight for over 6 h for PDMS to cure. The gap between the top and the bottom PDMS wells was thinly coated with PDMS two to three times to prevent leakage. When the platform was completely cured, the PVA tape was melted by immersing the platform in the heated DI for about 1 h.

### 4.5 Electrochemical Characterization

Electrochemical impedance spectroscopy (EIS) and cyclic voltammetry (CV) measurements were conducted using a potentiostat (Autolab PGSTAT302N, Metrohm AG, Switzerland) in a pH 7.4 PBS solution. The three‐electrode system was configured using the same electrodes as the PEDOT:PSS electrodeposition setup. For EIS, a 50‐mV sinusoidal signal was applied to each microelectrode, and the frequency was swept from 10 Hz to 1 MHz. For the CV, the potential was scanned from −0.6 to 0.8 V at 50 mV/s.

### 4.6 Generation of Human Heart Organoids (hHOs)

Human induced pluripotent stem cells (hiPSCs) were generated from BJ fibroblasts (ATCC) using episomal vectors, as previously described [30]. hiPSCs were maintained on Matrigel (Corning Inc.)‐coated dishes in mTeSR1 medium (STEMCELL Technologies Inc., Vancouver, BC, Canada) supplemented with 10 µm Y‐27632 (Tocris Bioscience). Medium was changed daily until cells reached ∼90% confluency. Cells were detached using DPBS containing 0.5 mm EDTA, collected in mTeSR1 supplemented with 10% Matrigel and 10 µm Y‐27632, and replated onto 60‐mm dishes. To induce embryoid body (EB) formation, cells were cultured in suspension on a shaker for 3 days with daily medium changes. The pieced EBs were then cultured for 2 days in RPMI1640 (Gibco) supplemented with B27 without insulin (B27(−); Gibco) and 6 µm CHIR99021 (Tocris Bioscience), followed by 2 days in RPMI1640+B27(−) containing 2 µm C‐59 (Selleckchem). From D7 of differentiation, medium was replaced with Advanced MEM (AD‐MEM; Gibco) supplemented with 1% GlutaMAX and penicillin/streptomycin (Gibco), refreshed every other day. Spontaneous beating of differentiated human heart organoids (hHOs) was typically observed around D10, and samples were collected on D20 for downstream analysis. This differentiation protocol was adapted from previously established methods [31, 32].

### 4.7 Immunofluorescence Staining of hHOs

D20 hHOs were fixed in 4% paraformaldehyde (PFA) at 4°C. Fixed samples were sequentially incubated in 15% and 30% sucrose solutions for 24 h each, followed by embedding in OCT compound. Cryosections (7–9 µm) were prepared using a cryostat at –20°C. Sections were permeabilized and blocked in PBS containing 0.1% Triton X‐100 (Sigma–Aldrich, USA) and 3% normal goat serum (NGS; Thermo Fisher Scientific) for 30 min at room temperature. Samples were incubated overnight at 4°C with primary antibodies (1:200) against cTnT (Abcam, ab45932), α‐actinin (Sigma–Aldrich, A7811), α‐smooth muscle actin (αSMA; Abcam, ab5694), and vimentin (VIM; Abcam, ab8978). After washing, sections were incubated for 2 h at room temperature with secondary antibodies (Alexa Fluor 488 goat anti‐mouse IgG and Alexa Fluor 594 goat anti‐rabbit IgG; 1:700, Invitrogen). Nuclei were counterstained with DAPI (1:1000; Thermo Fisher Scientific). Fluorescence images were acquired using a Nikon TE2000‐U microscope (Nikon). Color adjustments were applied to enhance visual distinction.

### 4.8 Generation of Induced Pluripotent Stem Cell (iPSC)‐Derived Cerebral Organoids

The human iPSC‐derived cerebral organoids were generated using the STEMdiff Cerebral Organoid Kit (08570 and 08571, StemCell Technologies) [33, 34, 35]. This study utilized the human iPSC line (BIONi010‐C) obtained from the European Bank for Induced Pluripotent Stem Cells. To generate embryoid bodies (EBs), iPSCs were detached by ReLeSR (100‐0483, StemCell Technologies) and then seeded into 96‐well clear round‐bottom ultralow attachment plates (7007, Corning) and maintained with the EB formation medium containing the ROCK inhibitor, Y‐27632, which was changed every two days without ROCK inhibitor. On day 5, EBs were transferred to the 24‐well clear flat‐bottom ultralow attachment plates (3473, Corning) in induction medium. On day 7, each EB was embedded in 15 µL of Matrigel hESC‐qualified matrix (354277, Corning) and transferred to a 6‐well clear flat‐bottom ultra‐low attachment plate (3471, Corning) in expansion medium. On day 10, the medium was changed to maturation medium. The maturation medium was replaced every 3 days until day 40 for maturation. After day 40, cerebral organoids were used for experiments. For using iPSCs and the generation of cerebral organoids, this project was approved by the Institutional Review Board of the Sungkyunkwan University (SKKU 2024‐10‐020).

### 4.9 Immunohistochemistry of Cerebral Organoids

Cerebral organoids were washed with DPBS and placed in 4% PFA at 4°C 24h. After 24 h, they were washed with DPBS to remove 4% PFA and put in 30% sucrose at 4°C for 48h, and transferred to a Biopsy cryomold (4565, SAKURA) and frozen in FSC 22 clear (3801480, Leica). The organoids were sectioned and mounted on slides. They were washed 3 times with DPBS, and permeabilized using 0.3% PBST for 30 min at room temperature (RT). Afterward, blocking was performed for 1 h at RT using 5% house serum (H0146, Sigma–Aldrich). Primary antibodies were diluted in 5% horse serum and incubated at 4°C overnight. The next day, primary antibodies were removed and washed 3 times with 0.3% PBST. Secondary antibodies were diluted at a 3% BAS in DPBS and incubated for 1 h at RT. Slides were washed 3 times with DPBS and DAPI were diluted in DPBS and incubated for 10 min at RT. Slides were washed 3 times with DPBS and mounted with cover glass. Primary antibodies used for immunohistochemistry were anti‐GFAP (1:500; 13‐0300, Invitrogen), anti‐MAP2 (1:500; ab5392, abcam) and anti‐PSD95 (1:500; MA1‐045, Invitrogen). Alexa Fluor 647/568/488 labeled secondary antibodies used 1:1000, and DAPI was diluted 1:5000 in DPBS. Images were acquired using a Leica Thunder Imager.

### 4.10 Live‐Dead Assay

Organoids were analyzed for viability using Cyto3D Live‐Dead assay kit (TheWell Bioscience, USA). The assay involved incubating the organoids in a 1:50 dilution of the Cyto3D reagent within the culture media at 37°C for a duration of 10 min. After incubation, the organoids were examined using a confocal microscope (LSM 980, ZEISS, Germany) to assess live and dead cells.

### 4.11 In Vitro Electrophysiological Recording

The packaged MN‐sMEA platform was sterilized by immersion in 70% ethanol for at least 30 min and then rinsed with sterile PBS. For heart organoid experiments, the rinse solution was replaced directly with heart organoid culture medium. For cerebral organoids, the device was immersed in 1% Matrigel (diluted in PBS; Corning, USA) and stored at 4°C overnight, after which the Matrigel solution was exchanged for cerebral organoid medium. Platforms containing culture medium were equilibrated in an incubator (NB203, N‐BIOTEK, Korea) at 37°C for ≥15 min before use. Organoids were transferred from the original culture plates onto the MN‐sMEA, and a 3D‐printed top pressor designed for the platform was positioned to secure the organoid in place. The assembled platform was returned to the incubator until recording.

For data acquisition, the amplifier board (RHD2000, Intan Technologies, USA) was connected to the platform via a cable routed into the incubator. Signals were sampled at 30 kHz with a hardware bandwidth of 1‐7500 Hz. Field potentials from heart organoids and multi‐unit spikes from cerebral organoids were processed using a custom spike‐sorting routine in MATLAB (MathWorks, USA). Prior to sorting, heart organoid recordings were band‐pass filtered between 1 and 300 Hz, whereas cerebral organoid recordings were filtered between 300 and 6000 Hz.

### 4.12 Pharmacological Treatments

Isoproterenol (Sigma–Aldrich, USA) and nifedipine (Sigma–Aldrich, USA) were dissolved in dimethyl sulfoxide (DMSO; Sigma–Aldrich). Each drug was prepared at 10× the working dose and diluted in AD‐MEM before use. The final DMSO dose was kept below 0.1%, which is considered non‐cytotoxic. PTZ (Sigma–Aldrich, USA) was dissolved in 0.22 µm filtered deionized water. PTZ was prepared at 10× the working dose and diluted in maturation medium before use.

### 4.13 Statistics

All results were performed using Origin (Originlab, USA) and presented as the mean ± standard deviation. Comparisons between more than three groups were performed using one‐way ANOVA, except for Figure 4g, which was analyzed using pairwise Welch *t*‐tests with Holm correction. Comparison between the two groups was performed using an unpaired *t*‐test. The specific levels of significance were indicated by asterisks: ^*^ (*p* < 0.05), ^**^ (*p* < 0.01), ^***^ (*p* <0.001), ^****^ (*p* <0.0001). n.s. stands for “Not significant”, which notes no statistical difference between the groups.

## Author Contributions

E.J., K.K., Y.K., and H.J.L contributed to the conceptualization of the study. E.J., S.S., S.G.L., K.K., Y.K., and H.J.L. contributed to the methodology. E.J., K.K., and H.J.L. developed the software. E.J., S.S., S.G.L., and J.S. conducted the investigation. E.J., S.S., and S.G.L. performed the visualization. I.J.C., J.C.P., C.Y.K., and H.J.L. provided resources. E.J., S.S., S.G.L., and J.A.G. contributed to validation. E.J. carried out formal analysis and data curation. H.J.L provided supervision. J.C.P. and H.J.L. contributed to funding acquisition. E.J., S.S., S.G.L., and H.J.L. prepared the original draft of the manuscript. E.J., S.S., S.G.L., K.K., Y.K., J.S., I.J.C., J.A.G., J.C.P., C.Y.K., and H.J.L. contributed to writing through review and editing.

## Conflicts of Interest

The authors declare no conflict of interest.

## Supporting information




**Supporting File**: advs75778‐sup‐0001‐SuppMat.docx.

## Data Availability

The data that support the findings of this study are available from the corresponding author upon request.
