# Supplementary material for: Stretchable Microelectrode Arrays with Microneedles for Reliable Electrophysiological Recording of Human Heart and Brain Organoids
Source: Adv Sci (Weinh). 2026 May 20:e75778. Online ahead of print. doi: 10.1002/advs.75778 (PMC13335954; doi:10.1002/advs.75778)
Supplement: Supplementary file 1 — Supporting File: advs75778‐sup‐0001‐SuppMat.docx. [file ADVS-9999-e75778-s001.docx]

Supporting Information

Stretchable microelectrode arrays with microneedles for reliable electrophysiological recording of human heart and brain organoids

Eunyoung Jang ^†^, Saewoon Shin^†^, Seul-Gi Lee ^†^, Kiup Kim, Yoojeong Kim, Jun Sun, Il-Joo Cho, Joseph A. Gogos, Jong-Chan Park^*^, C-Yoon Kim^*^, and Hyunjoo J. Lee^*^

**Supplementary Figures**


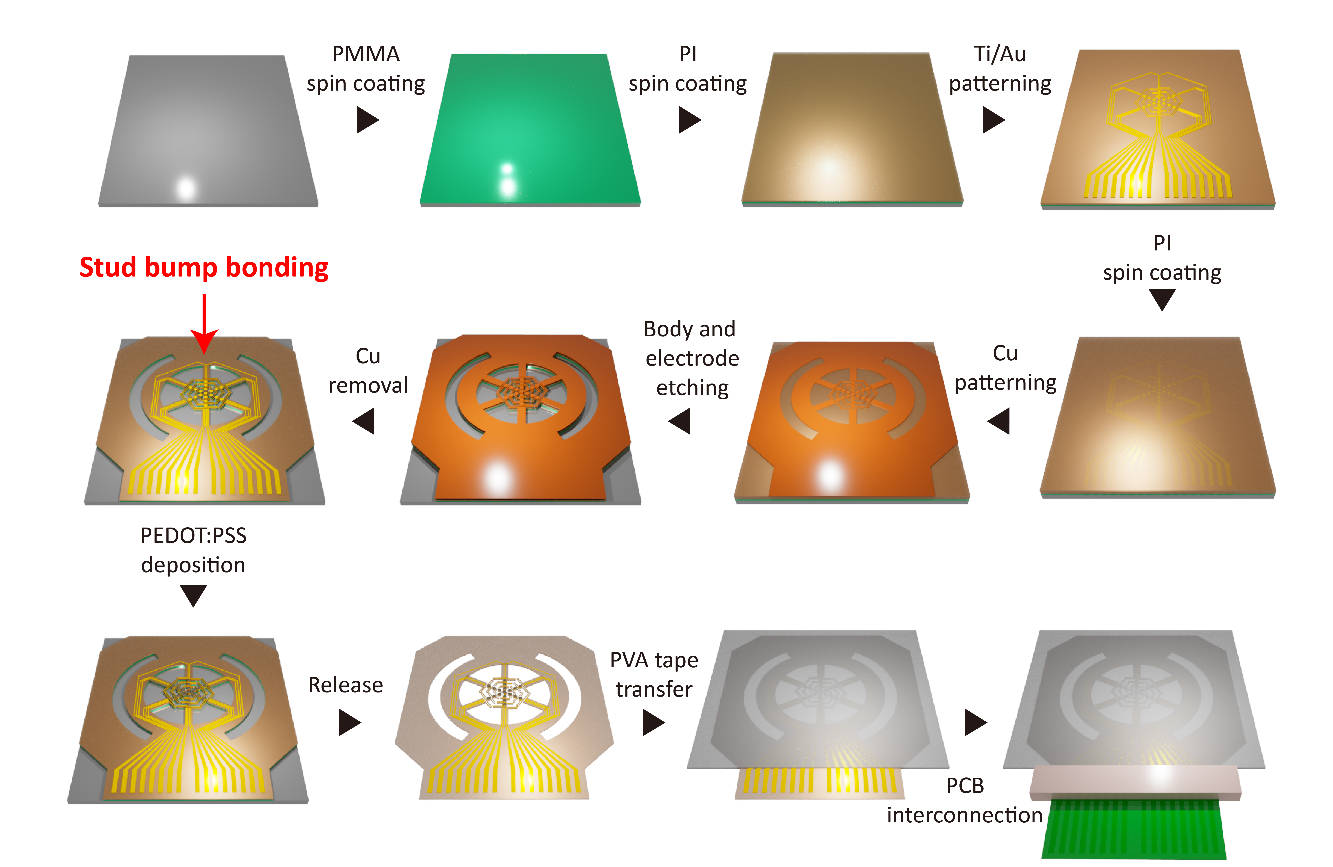


**Figure S1.** Schematic illustration of MEMS fabrication process of stretchable MEA.


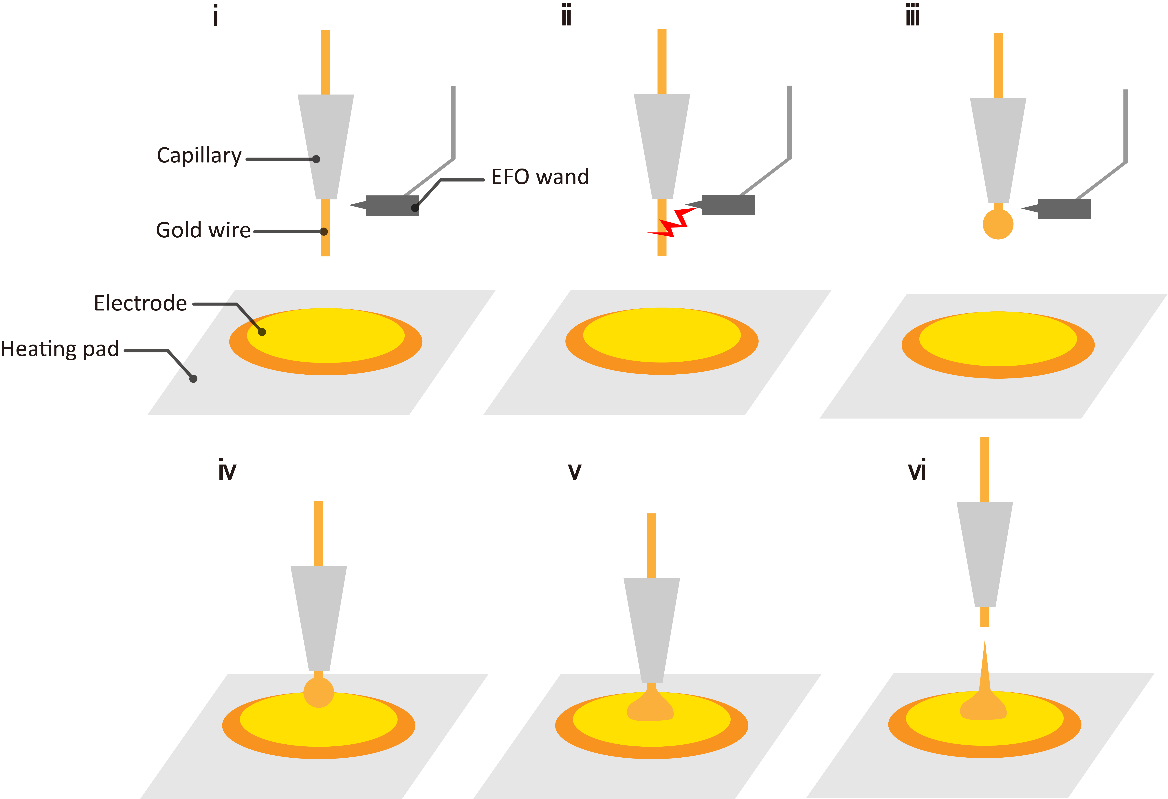


**Figure S2.** Schematic illustration of the gold stud bump process to form a microneedle on a planar microelectrode. (ⅰ) Positioning of the capillary containing the gold wire over the target planar microelectrode. (ⅱ, ⅲ) Formation of a gold ball at the end of the wire using an electronic flame-off (EFO) wand. (ⅳ) Descending the capillary to bring the gold ball into contact with the microelectrode surface. (ⅴ) Upward movement of the capillary while maintaining wire tension. (ⅵ) Tearing of the gold wire to complete the formation of microneedle on the planar microelectrode.


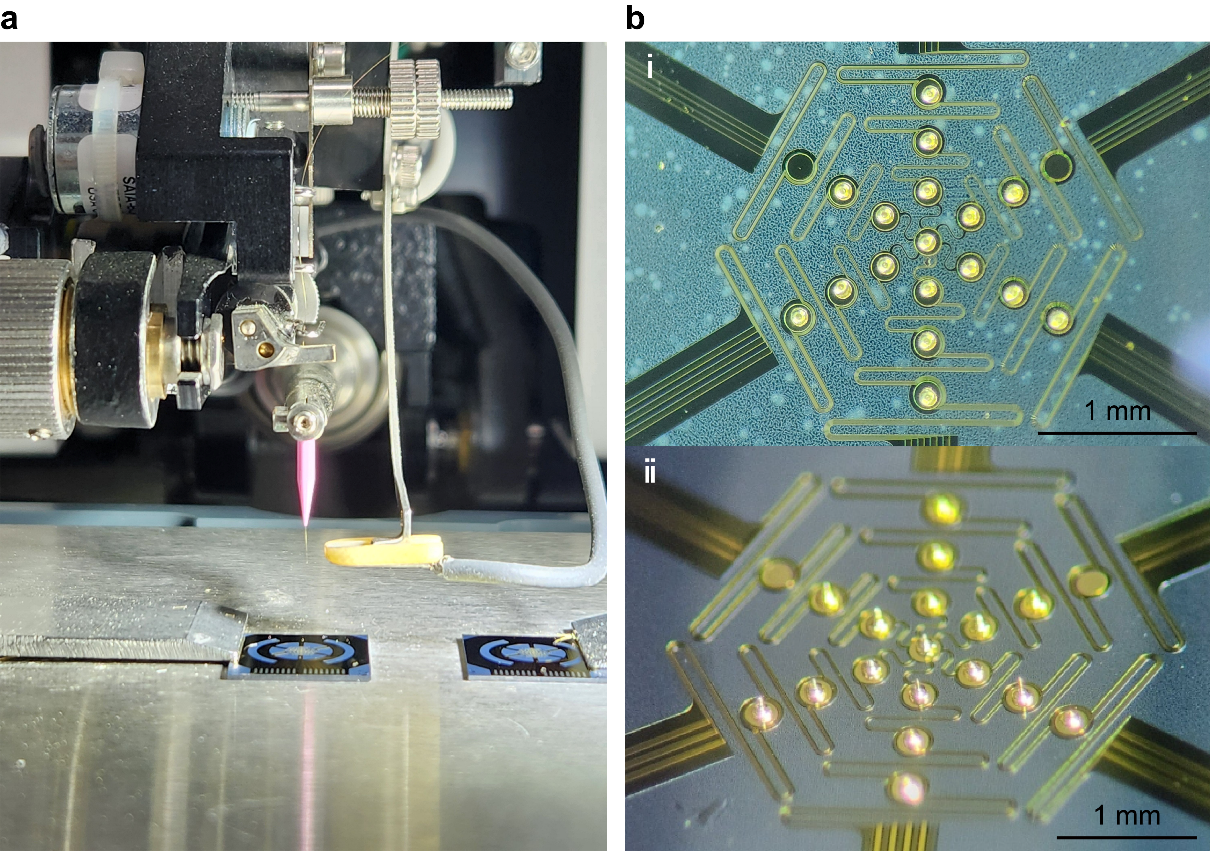


**Figure S3.** (a) A photograph of wire bonding set up for forming microneedles on top of the fabricated sMEA. (b) Optical images of the microneedle sMEA: (ⅰ) top view and (ⅱ) tilted view.


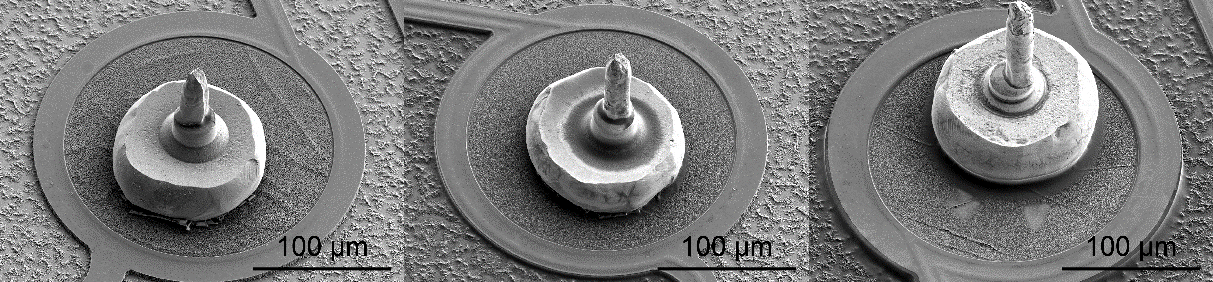


**Figure S4.** Tilted-view SEM images of a single Au microneedle fabricated on microelectrodes via stud bump process.

**
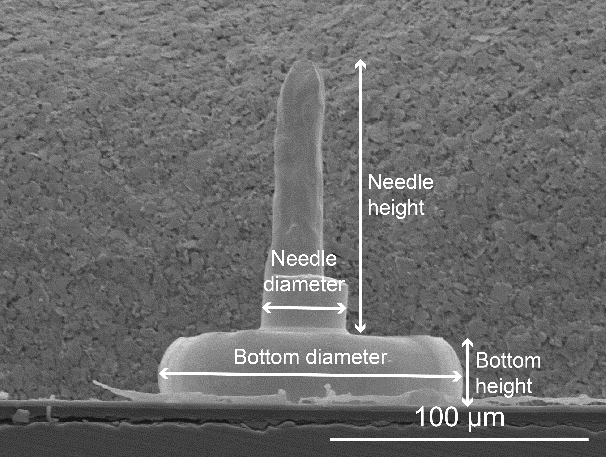
**

**Figure S5.** Representative SEM image illustrates the measurement criteria used for quantitative analysis of the microneedle geometry.


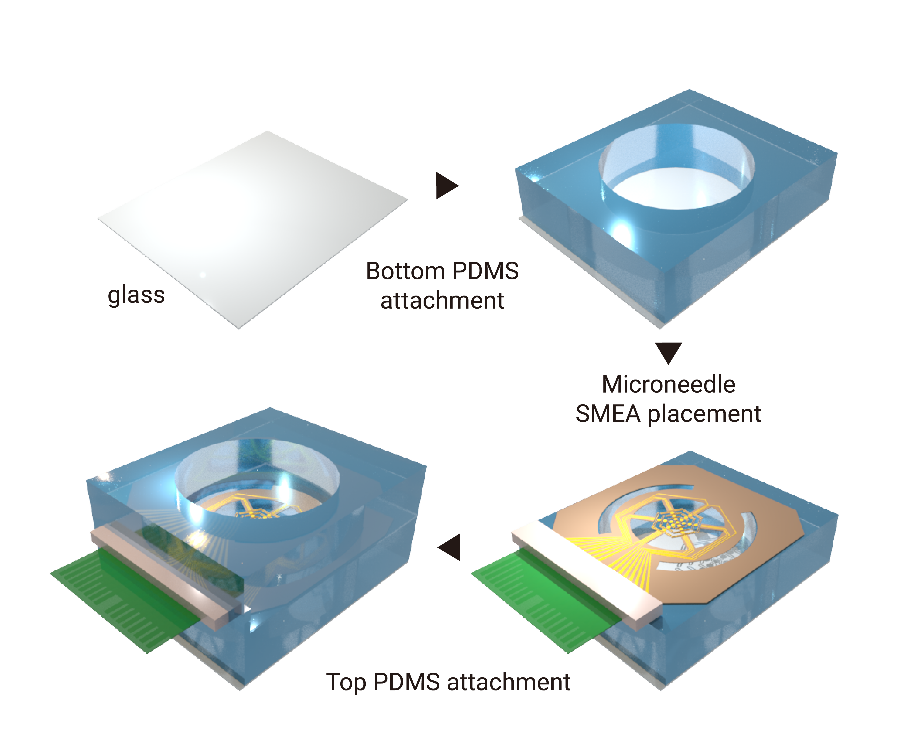


**Figure S6.** Schematic illustration of the packaging process of 3D-MN-sMEA platform.


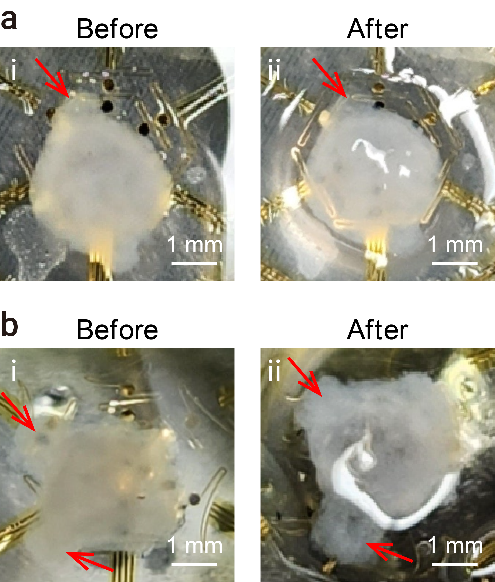


**Figure S7**. Morphological comparison of organoids on 2D and 3D platforms. (a) Optical images of a heart organoid on conventional 2D surface before (ⅰ) and after (ⅱ) lowering the medium level to enhance contact. (b) Optical images of an organoid on the 3D MN-sMEA before (ⅰ) and after (ⅱ) similar adjustments.


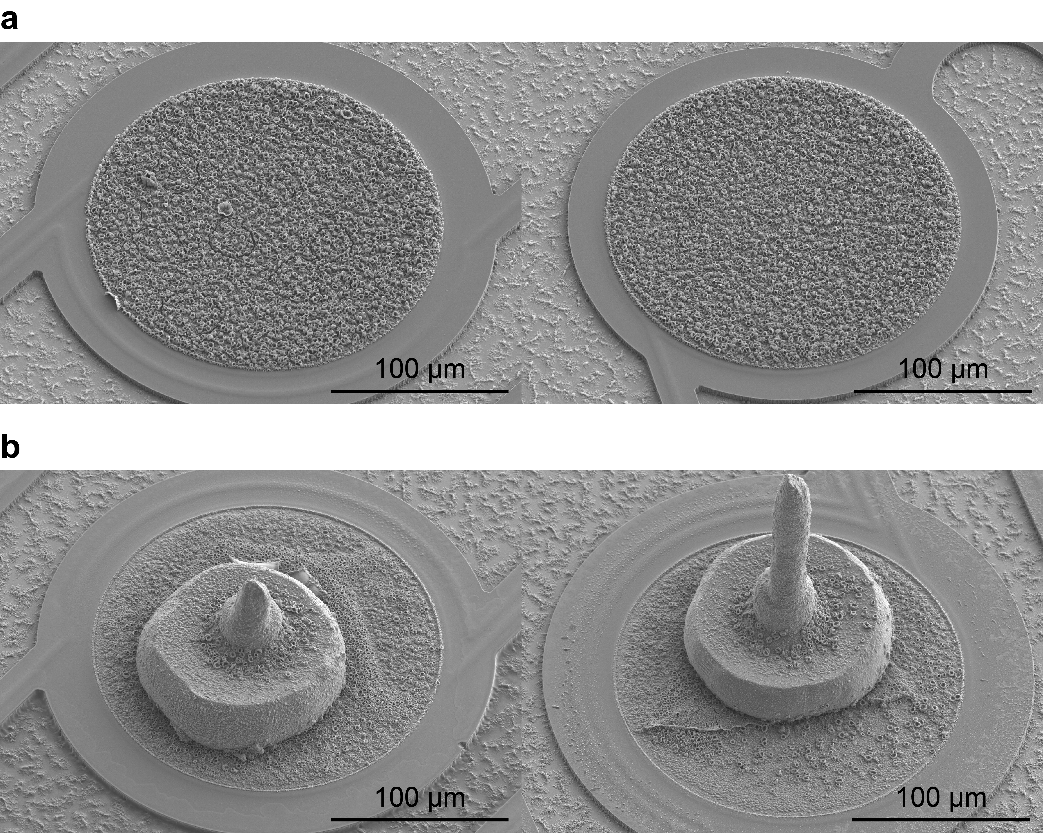


**Figure S8.** SEM images of the (a) PEDOT:PSS electrodeposited planar microelectrodes and (b) PEDOT:PSS electrodeposited microneedle microelectrodes (PEDOT:PSS MN-ME).

**
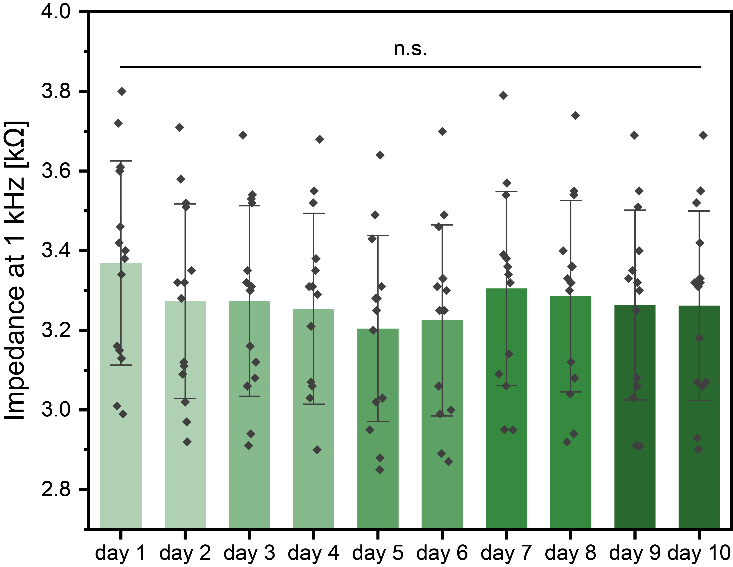
**

**Figure S9**. Impedance magnitude at 1 kHz measured in PBS (pH 7.4) inside 37℃ CO_2_ incubator over a 10-day period (*n* = 14). Data are presented as mean ± SD. n.s., not significant.


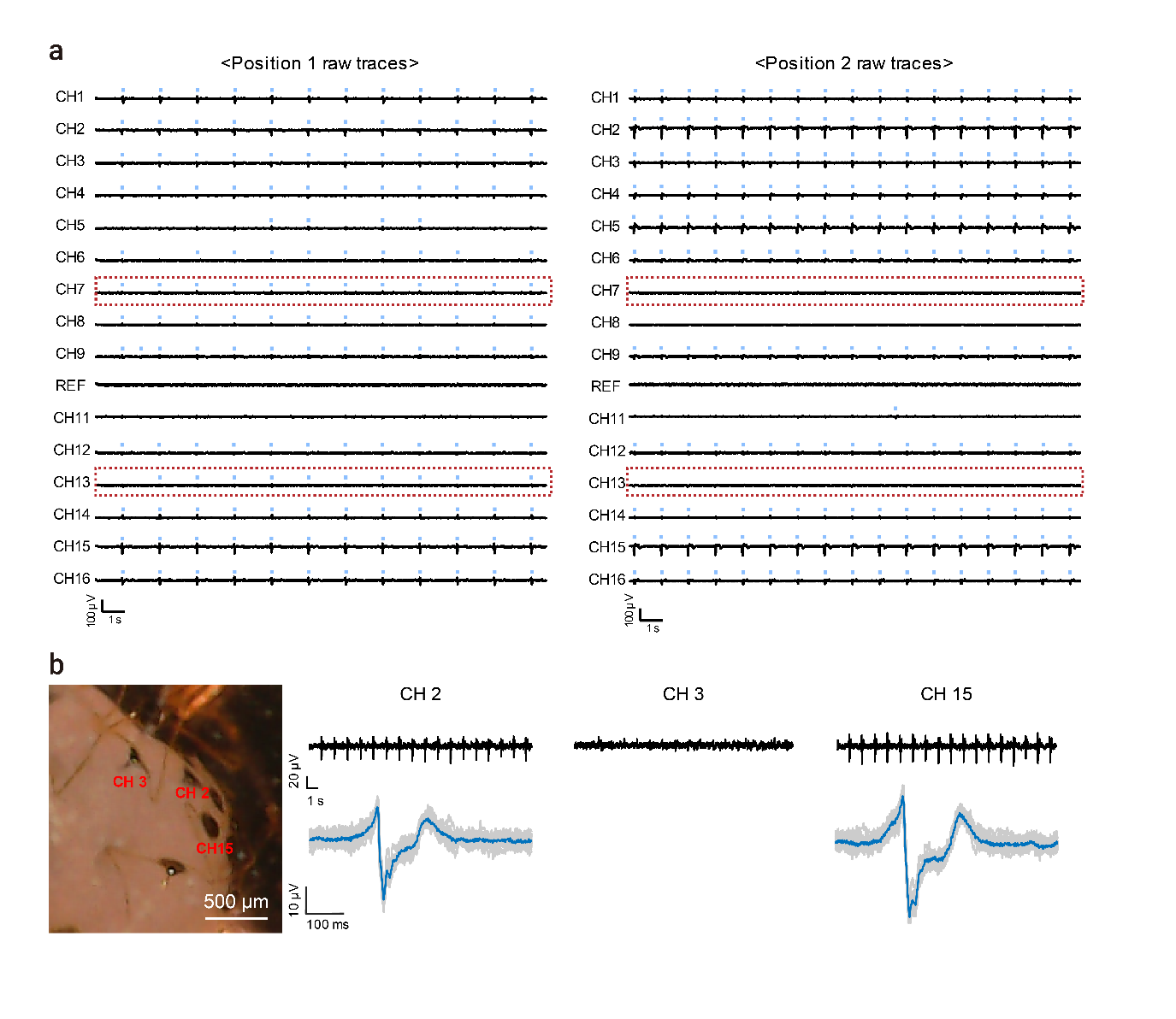


**Figure S10**. Qualitative analysis of the correlation between penetration depth and signal quality. (a) Raw field potential traces according to organoid orientation from position 1 to position 2. (b) Optical verification of the needle-tissue interface and raw traces and average field potential of CH 2, CH 3 and CH15, respectively.


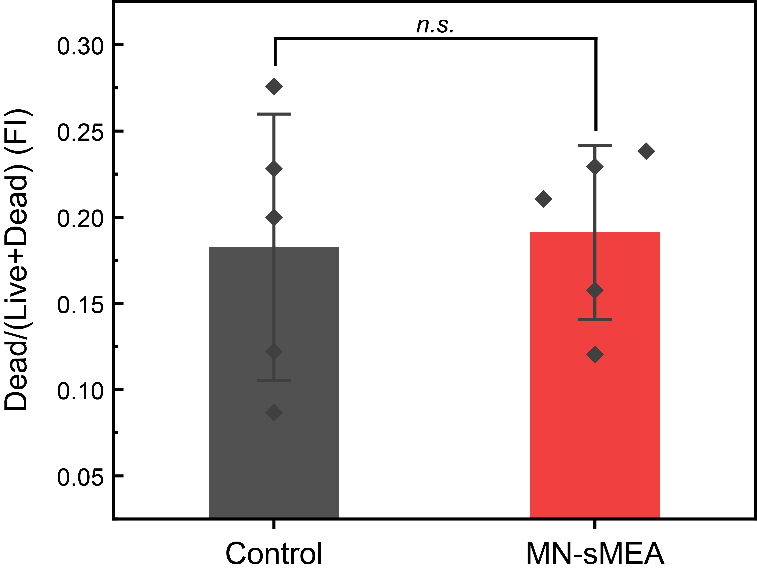


**Figure S11.** Live-dead assay of cerebral organoids cultured on culture dish (control) and 3D microneedle sMEA (MN-sMEA) demonstrating the biocompatibility of 3D MN-sMEA. Data are presented as mean ± SD. n.s., not significant.


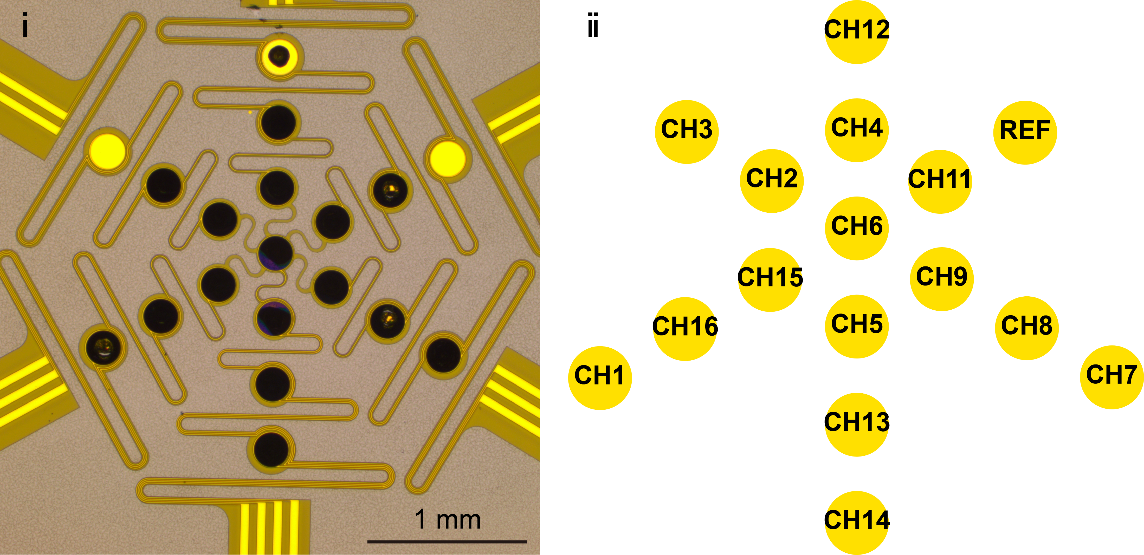


**Figure S12.** (ⅰ) An optical image of PEDOT:PSS electrodeposited microneedle sMEA and (ⅱ) the corresponding channel numbers.


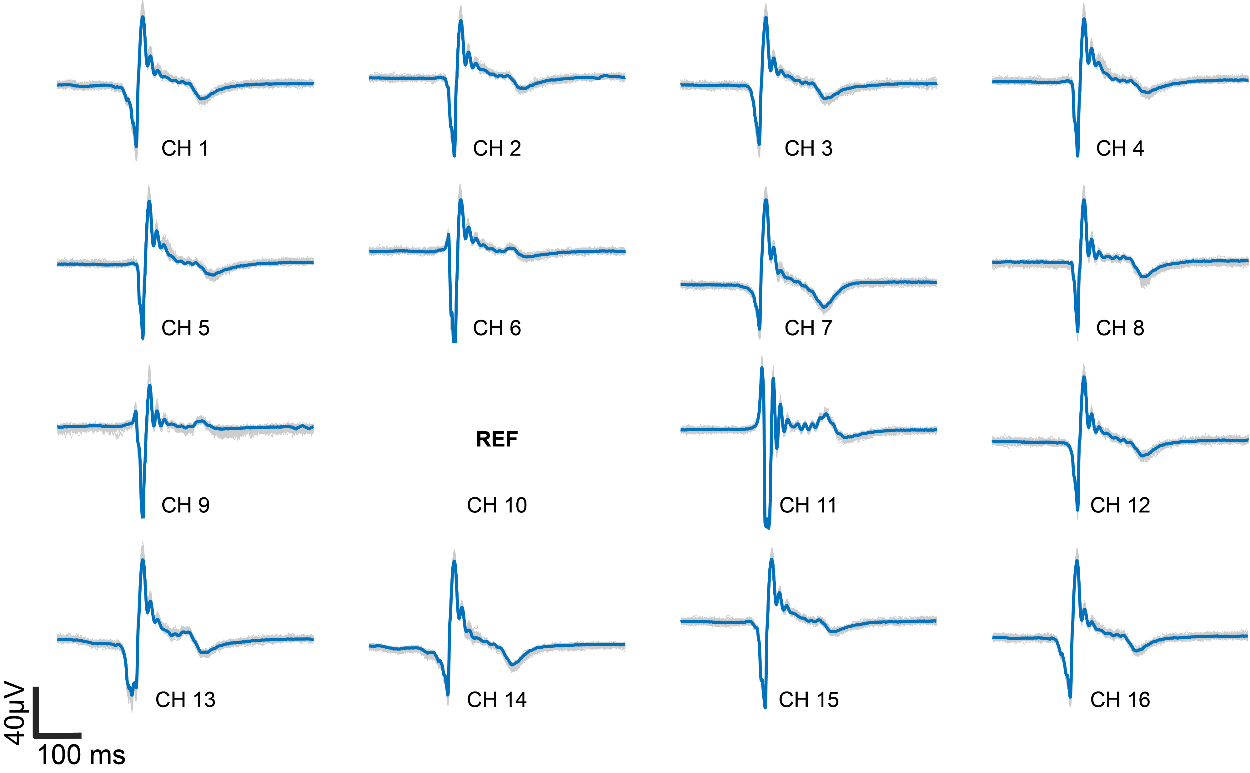


**Figure S13.** Averaged field potential waveforms from all channels.

| **Substrate** | **Microneedle** | **Pros** | **Cons** |
| --- | --- | --- | --- |
| Flexible | Flexible | - Minimal organoid deformation - Minimal tissue damage | - Reduced electrode stability due to needle deformation - Decreased signal reliability |
|  | **Rigid**  **(This work)** | - Minimal organoid deformation - Maximized electrode stability - Stable interface during contraction/relaxation | - Minimal tissue damage from needles |
| Rigid | Flexible | - Minimal tissue damage | - Significant organoid deformation - Reduced electrode stability - Unstable signal acquisition |
|  | Rigid | - - | - Maximal organoid deformation - Maximal tissue damage - High risk of interface failure during movement |

**Table S1**. Qualitative comparison of different substrate and microneedle combinations for heart organoid monitoring.
